# Advanced Driver Assistance Systems (ADAS) Based on Machine Learning Techniques for the Detection and Transcription of Variable Message Signs on Roads

**DOI:** 10.3390/s21175866

**Published:** 2021-08-31

**Authors:** Gonzalo De-Las-Heras, Javier Sánchez-Soriano, Enrique Puertas

**Affiliations:** 1 SICE Canada Inc., Toronto, ON M4P 1G8, Canada; gdelasheras@sice.com; 2 Department of Science, Computing and Technology Department, Universidad Europea de Madrid, 28670 Madrid, Spain; javier.sanchez@universidadeuropea.es

**Keywords:** VMS, machine learning, ADAS, image processing, environment perception

## Abstract

Among the reasons for traffic accidents, distractions are the most common. Although there are many traffic signs on the road that contribute to safety, variable message signs (VMSs) require special attention, which is transformed into distraction. ADAS (advanced driver assistance system) devices are advanced systems that perceive the environment and provide assistance to the driver for his comfort or safety. This project aims to develop a prototype of a VMS (variable message sign) reading system using machine learning techniques, which are still not used, especially in this aspect. The assistant consists of two parts: a first one that recognizes the signal on the street and another one that extracts its text and transforms it into speech. For the first one, a set of images were labeled in PASCAL VOC format by manual annotations, scraping and data augmentation. With this dataset, the VMS recognition model was trained, a RetinaNet based off of ResNet50 pretrained on the dataset COCO. Firstly, in the reading process, the images were preprocessed and binarized to achieve the best possible quality. Finally, the extraction was done by the Tesseract OCR model in its 4.0 version, and the speech was done by the cloud service of IBM Watson Text to Speech.

## 1. Introduction

### 1.1. Motivation

Since the democratization of the private car, the world’s fleet has continued to grow [1,2] (in Spain, each household has almost two vehicles [3]). This increase has brought with it the problem of traffic accidents. Data from the World Health Organization (WHO) estimate that during the period 2011–2020, 1.1 million people died due to traffic accidents and between 20 and 50 million were injured [4].

In Spain, the Dirección General de Tráfico (DGT) has produced a series of statistical yearbooks, which illustrate the evolution from 1960 to 2018 [5,6]. Generally speaking, the number of casualties has increased in recent years. The number of fatalities and hospitalized victims has decreased while the number of non-hospitalized injured victims has increased. Accidents are still occurring, but the probability of death is decreasing.

The causes of traffic accidents can be classified according to the risk factor that causes them. They are distinguished by human, mechanical and environmental factors (the state of the asphalt or traffic signs and weather conditions). According to the DGT, in 2018, 88% of accidents were the result of inappropriate driver behaviors [7] (similar conclusion to study [8], which states that 90% are due to human causes). In first place were distractions (33%), followed by speeding (29%) and alcohol consumption (26%) [7]. The same organization has prepared a document that lists the main distractions and explains how they affect accidents [9]. It shows that actions such as using a cell phone, eating or smoking are activities that require time and attention, reducing concentration while driving. The driver’s physical condition also affects his reaction time and ability to be distracted. This has a direct impact on braking distance, which is a serious risk. Many of these behaviors are known to drivers and many are declared offenders [10].

The WHO, in its report on 2011–20, proposes five action points to improve safety [4]. Examples of the third (safer vehicles) are initiatives such as Prometheus [11,12], created by an association of vehicle manufacturers and researchers, or DRIVE (Dedicated Road Infrastructure for Vehicle Safety in Europe), funded by the EU (European Union) [12], which has promulgated a large number of papers on fundamental and practical problems, such as GIDS (Generic Intelligent Driver Support) [13]. Its aim was to “to determine the requirements and design standards for a class of intelligent co-driver (GIDS) systems that are maximally consistent with the information requirements and performance capabilities of the human driver” [13]. It was the beginning of what we know today as ADASs (advanced driver assistance systems), successors to basic safety systems and enablers of autonomous driving in the future [14].

**Figure 1:**
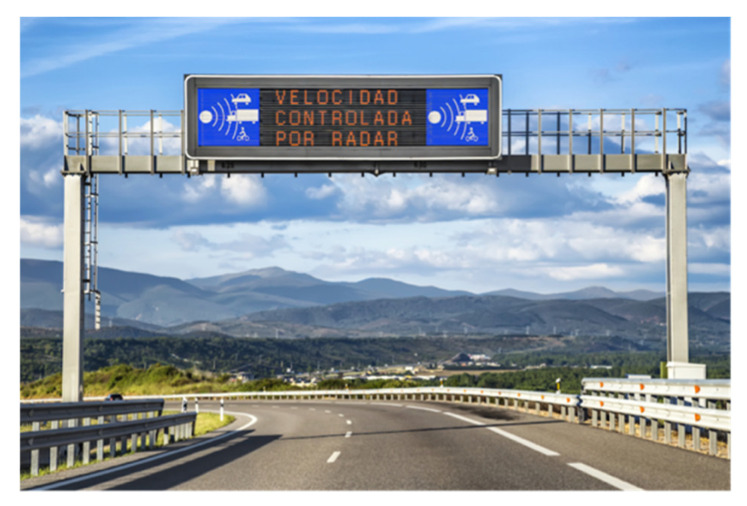
VMS example [16].

Variable message signs (VMSs) are roadside ATIS (advanced traveler information system) devices consisting of LEDs (light-emitting diodes) that stand out against a black background (Figure 1). They are the mechanism used by traffic agencies to communicate useful information to drivers in order to improve their safety. These messages convey information by means of personalized text and/or traffic sign pictograms [15].

Several studies indicate that VMSs have a positive impact on driving by reducing speed [17] and relieving congestion caused by accidents or other events [18]. The very act of reading the VMS itself causes a reduction in speed while approaching it [19]. However, the act of investing attention and time into reading the message and understanding it is in itself a distraction and therefore a risk. Additionally, if we add to a main task, such as driving, the task of reading and understanding the information, we obtain a decrease in the effectiveness of both tasks [20]. There are approaches to reduce the attention required, simplifying the information by means of pictograms or messages consisting of a single word. The latter are more effective in understanding the message than even pictograms, because comprehension does not depend on prior knowledge of the pictogram [21]. There are conventions, such as the Vienna Convention [22], but each country is free to alter their signs, which makes it difficult to recognize them quickly.

There are solutions such as READit VMS [23], which through a client–server architecture and the geolocation of the user performs a locution of the content of the sign or displays a pictogram on an internal screen of the vehicle. These applications require constant connectivity to geolocation and the Internet to check the nearest VMS and may suffer from latency issues. They are also limited to the VMSs registered in the system. Due to these dependencies, they are not autonomous systems that allow the vehicle to be independent wherever it travels. The most similar ADAS are traffic signal recognition systems that, using sophisticated computer vision and machine learning techniques, display the signal to the driver on a screen located on the dashboard.

The motivation of this project is to provide solutions to the challenge of road fatalities by developing an ADAS that intervenes in the major cause of accidents, the distractions [7,10]. On the road we find panels with information that many studies have reported to cause a reduction in vehicle speed. However, the cause of this is the attention that is required to read and understand the message [24]. This results in less efficient driving [20]. This issue has been addressed by client–server software [23], but not by machine learning and computer vision techniques. This ADAS will allow the vehicle to be independent from network latency, geopositioning and the sign database. The solution will consist of a VMS recognizer that reproduces the signal content using a synthetic voice. To do so, it recognizes and trims the VMS from the road images, delivers it to the OCR (optical character recognition) subsystem that transcribes the panel content and announces it via the IBM Watson Text to Speech cloud service [25].

### 1.2. Vehicle Safety Systems

The report [14] carried out by The Boston Consulting Group (BCG) for The Motor & Equipment Manufacturers Association (MEMA) describes the evolution of safety systems in three periods: assistance and comfort systems, ADASs and semi/autonomous vehicles.

**First assistants.** In the first period, the first projects were developed to improve vehicle safety. Although they may seem simple, they are very useful, since they not only help the driver, but also provide greater comfort (an aspect closely related to safety [26]).

Some of these systems are cruise control, ABS (antilock braking system), ESP (electronic stability program), etc.

**ADAS.** As technology developed, more advanced systems emerged that operated in increasingly complex situations. The report [27] proposes a taxonomy based on the type of sensor used:

- Vision systems. These have cameras (monocular, stereo and infrared) placed at strategic points of the vehicle that provide images of the environment from which knowledge of the scene is extracted. These kinds of systems have problems with depth and lens obstructions; however, they are affordable [27];
- LiDAR (light detection and ranging). This is a technology that generates a 3D environment by projecting rays and measuring the distance to different objects. This allows the vehicle to know the elements around it in high resolution. It is a cutting-edge technology, but at the same time expensive. There is currently a debate between LiDAR and conventional cameras. Companies on a par with Tesla bet on the extraction of knowledge through multiple cameras plus other devices, such as radars. Others, for instance Waymo, believe that LiDAR is the solution of the future [28];
- Radars. These systems measure the speed and distance of objects in the environment (thanks to the Doppler effect). They emit a series of microwaves and measure the change in wave frequency. One case of use is adaptive cruise control [27];
- Ultrasound. Using a series of sound waves, these systems measure the distance to nearby objects. An example is the parking collision warning device [27];
- All these ADASs are complemented with other functionalities to improve their accuracy. For example, IMUs (inertial measurement units) or GPSs (global positioning systems) are auxiliary systems for distance measurement [27].

**Semi/autonomous vehicles.** In the latest era, which comes up to the present day, the challenge is to create cars that can drive themselves. With the help of new ADAS, such as the autopilot for traffic jams or the automatic lane change, this is possible. By 2025, it is expected that there will be 8 million autonomous and semi-autonomous vehicles worldwide [29,30,31].

The J3016 standard “Levels of Driving Automation” of the Society of Automotive Engineers (SAE) established six levels with which to define the autonomy of a vehicle. They range from 0 (fully manual) to 5 (fully autonomous) [32].

### 1.3. Recognition Systems

#### 1.3.1. Object Recognition

The history of object recognizers is divided into two periods: traditional models and, since 2014, those based on deep learning [33].

First-generation detectors had to deal with a lack of computational and feature representation resources. For this reason, these algorithms contained hand-crafted features and methods that took full advantage of machine power [33].

- **Viola Jones** [34,35]. This is an extremely fast face recognizer, which slides a window over the entire image until a face is identified in one of the subsections.
- **HOG (histogram of oriented gradients)** [36]. This detector is designed to work on a uniform grid. Although it can be used to detect a variety of objects, it was primarily motivated for pedestrian detection [33].
- **DPM (deformable part-based model)** [37]. This method is an extension of the HOG detector, which applies the divide and conquer strategy. For example, the problem of recognizing a car can be decomposed into locating parts such as wheels or windows. It consists of a main filter and several secondary filters configured by supervised learning as if they were latent variables [33].

With the evolution of machine learning techniques, artificial neural networks (ANNs) emerged and within them, deep convolutional neural networks (CNNs) have improved image classification [38,39] and object detection [39,40,41] accuracy. Within CNNs, those dedicated to object detection are divided into two groups: one-stage and two-stage. The first ones treat the task as a regression problem by learning the probabilities of a class and the coordinates of the bounding box. The second ones group a series of regions of interest (first step) that are sent to the object classifier and the coordinate delimiter (second step). Each strategy has advantages and disadvantages. For example, one-step ones are faster, but have less accuracy [42].

Two-stage models:

- **R-CNN** [40]. This system takes the image and divides it into about 2000 regions on which the features are computed by a CNN. Finally, each region is classified by linear one-vs-rest SVMs (support vector machines) [40];
- **Fast R-CNN** [39]. Based on the previous model, fast R-CNN directly extracts features from the entire image, which are sent to the CNN for classification and localization at the same time. Thanks to this improvement, training time decreases while accuracy increases [39];
- **Faster R-CNN** [43]. This model eliminates the bottleneck that fast R-CNN had when selecting the region of interest (RoI) [33] by using a CNN called a region proposal network (RPN) to predict it. Faster R-CNN merges the RPN and fast R-CNN into a single network, so that the first one tells the second one where to focus. This is achieved by sharing their convolutional characteristics. This way, the RoI selection is practically zero cost, and the system is very close to real time [43].

Single-stage models:

- **YOLO** (You Only Look Once v1 [44], v2/9000 [45], v3 [46], v4 [47]). This is a real-time object recognition system thanks to the fact that the entire detection process is done by a single network. The process consists of a phase in which the system resizes the image to 488 x 488 and then executes a single CNN that returns the confidence of the detected object [44]. There are several enhancements to this model that are focused on increasing the accuracy but keeping the fast execution. The most recent version is v4 [45,46,47];
- **SSD (single shot detector)** [48]. This model’s main contribution is the introduction of multi-reference and multi-resolution detection techniques, which significantly improve detection accuracy, especially for some small objects [33];
- **RetinaNet** [49]. Thanks to the authors of [49], it was found that the extreme imbalance of the foreground class is the main cause of their lower accuracy. To solve it, they introduced a new loss function called "focal loss" to make the classifier focus on the most difficult examples of the misclassified ones. This brings this model up to the accuracy of the two-stage models.

There are several surveys in the literature that compare these object recognition models by measuring accuracy and speed, both for training and for inference. One of the best works comparing each of these models is [50], in which a systematic review of each of the models presented above is made and they are compared in terms of different metrics such as accuracy or inference speed. It is difficult to choose a clear winner since it depends on the specific task we are performing and whether we are more interested in a fast model for inference or if we need to obtain a higher accuracy in object recognition. In our work we have chosen RetinaNet as it is a model with one of the best accuracy–FPS balances.

#### 1.3.2. Text Recognition

As with object detection, there are two eras. A first one in which the techniques were based on “hand-made” features to discriminate the characters, and another one in which machine learning models predominate [51,52].

Pre-deep learning period:

- **Connected-component analysis (CCA).** These classifiers extract candidate components at first and then filter out non-textual components using manual rules or trained classifiers [53]. There are two methods, these being stroke width transform (SWT) and maximally stable extremal regions (MSER) [51];
- **Sliding window (SW).** This model works by sliding a small multi-scale window through all possible locations on the image, classifying whether text is present or not [51].

In the era of deep learning, [52] proposes a hierarchical taxonomy divided into text detectors, transcribers, end-to-end systems and auxiliary methods that improve the model quality:

- **Detection.** Text detection can be defined as a subset of the problem of object detection, in which there are three tendencies [52]:
- **Reduction of pipelines** to simplify the training process and reduce error. **Decomposition into subtexts** and then joining them into a complete instance. **Specific recognition** in cases such as curved text, irregularly shaped text or text with complex backgrounds;
- **Transcribers.** In traditional methods, the process consisted of preprocessing, segmentation and character recognition. However, segmentation is costly and has a longer execution time. To avoid this step, connectionist temporal classification (CCT) methods [54] and attention mechanisms [52] are used;
- **End-to-end systems.** Instead of dividing the main problem into detection and recognition subproblems, these systems integrate the entire process for reading directly from the image [52];
- **Auxiliary techniques.** An important aspect is techniques that improve training quality, such as creating synthetic examples, reducing noise in the image or incorporating information from the environment [52].

Some examples for object detection in vehicle security systems are:

Traffic light recognition [55,56,57]. These are assistants that detect this type of signaling, so that they can inform the driver of their current status. If they were connected directly to the vehicle control system, the vehicle could even brake automatically. The main challenges of this ADAS are related to the different types of traffic lights, since there are several models depending on the country, and the existence of intersections or multiple lanes;

Signal recognition [58,59]. Traffic sign identification is one of the tasks required for environment perception. They are the main source through which drivers receive information (maximum speed, prohibitions, intersections, etc.). Although there are currently commercialized ADAS (such as the Toyota Road Sign Assist, or RSA [60]), it is still a challenge. The main problem is the diversity in size and shapes;

Panel recognition [61,62]. Information boards are a type of signage located above the lanes, which primarily communicate information by text. Therefore, the challenge for the assistants lies in the recognition of the characters, not only in the identification of the object on the road.

## 2. Methodology

**Figure 2:**
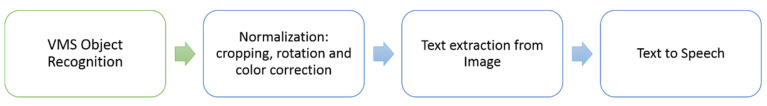
Processing steps.

The processing steps are summarized in Figure 2. The images captured by the vehicle camera are initially processed by the VMS object recognition module. The next step is to normalize the section that corresponds to the VMS by cropping the image, changing the perspective and angle in addition to adjusting the color to facilitate the following task of extracting the text from the image. Finally, the text is converted to audio using a “text to speech” service in the cloud.

**Figure 3:**
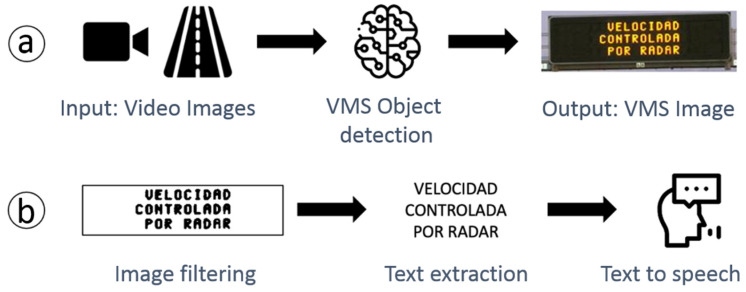
The VMS reading process consists of 2 steps. (**a**) VMS extraction and (**b**) processes the image to extract the content and speak it.

These processing steps for the VMS speech system are divided into two subsystems combining local processing and cloud services: a VMS recognizer and a content extractor and speaker (Figure 3).

### 2.1. VMS Recognizer

From a picture of the environment taken by a camera located on the front of the vehicle, it recognizes the VMS and produces another image as an output, consisting only of the sign itself. This task is carried out by a deep CNN, a machine learning model that gives great results in image classification and object detection [38,39,40,41]. In order to do so, it is necessary to build a set of labeled images to train and evaluate the model.

### 2.2. Content Extractor and Speaker

Taking as an input the image produced by the VMS recognizer, it processes it to obtain the text of the panel and reproduces it using a synthetic voice. The process is as follows.

First, it is necessary to preprocess the image to make it easier to extract the text. The steps to follow are: (1) Angle correction. Straightens the orientation of the VMS. (2) Cropping of the VMS. Generates an image with only the content of the panel by eliminating margins that do not correspond to the VMS. (3) Color adjustment. Transforms the previous image into another one with black text over a white background; this will make the extraction task easier.

Then, using an OCR model, it transcribes the text contained in the panel. Finally, the system makes a call to the IBM Watson Text to Speech cloud service, which returns a sound file with the spoken text.

## 3. Variable Message Sign Recognition

### 3.1. Dataset

#### Labeled Image Collection

The strategy is to join different sources to maximize the number of examples with the least manual work. This is a key point, since each image must be annotated individually, which is very time-consuming. Therefore, a process has been designed to obtain a minimal dataset and to create a basic model with which to label the images iteratively. Thus, although the first search will be completely manual, subsequent searches will consist of small adjustments on images extracted from videos (Table A1), which would otherwise involve a lot of work. The initial acquisition can be divided into three steps:

1. **Collection.** By searching Google Images, YouTube, several websites and manual clippings combined with scraping scripts.
2. **Labeling.** Each image is manually annotated using the software in [63], which generates an XML (Extensible Markup Language) file in PASCAL VOC (Visual Object Classes) format.
3. **Data augmentation.** Data augmentation is a widespread method that consists of applying modifications to the image (rotations, cropping, translations, etc.) in order to create apparently new instances. For this project, since the VMS will always be in the top position of the image, we have chosen to flip the image on the y-axis. That way, the signs on one side will be placed on the opposite side, generating a new instance.

Once the first version of the dataset (134 VMS examples) was obtained, a RetinaNet [49] was trained with it on a ResNet50 model [64] pretrained on COCO [65]. This model has been selected due to the fact that even though it is a single-stage model, it achieves results very close to those of two stages, maintaining the advantages of the single-stage models [49]. Results are shown on Table 1.

Thanks to this model, an iterative process begins in which new labeled images are obtained more quickly. There are two methods with which to do so:

- **Manual.** As in the first acquisition, the VMS images are manually selected. The difference is that the labeling is performed by the basic model;
- **Semiautomatic.** In this case, we select videos to be analyzed by the basic model in order to extract a set of labeled candidate images from hours of footage, which would otherwise be much more tedious.

Since this first model is not perfect (nor is it intended to be), it is necessary to check the automatic selection and detection. Finally, once the images have been validated with their annotations, data augmentation (flipping on the *y*-axis) is applied.

### 3.2. Final Dataset

Every machine learning algorithm is sensitive to overfitting its parameters to the data with which it has been trained. In this situation, the model memorizes this information, which prevents it from generalizing and, therefore, from performing well in real situations. To avoid this situation, the dataset has been divided into two portions, one exclusively for training and another for validation. This method is a popular practice for correctly measuring the quality of a model.

At a certain epoch, generalization is transformed into memorization of the training set. This manifests itself as an increase in the validation error after a downward trend, while the training error decreases until it almost disappears. The best model is found just before this occurs.

The training set contains 706 (324 with VMSs) images extracted partially from 19 YouTube videos with a total duration of 05:19:27. The test set contains 153 (56 with VMSs) images that were manually reviewed to ensure the best comparison.

### 3.3. VMS Recognizer

Next, the training process performed to obtain the final model is detailed. A public distribution called Keras RetinaNet [66] has been used, which works on TensorFlow 2.0 [67]. Table 2 shows hardware specifications of on-board PC used for training and deployment.

It has been established as an indicator to maximize the AP (average precision), which is the area under the coverage–precision curve given an IoU (Intersection over Union). The IoU indicates the amount of overlap between the recognized area and the real area. It is used as a threshold to find the true positives (TP), false positives (FP) and false negatives (FN) that define the accuracy and coverage value.

The training parameters and results (Table 3) are as follows.

Once the first training is finished, it can be resumed by reducing the learning rate (lr) to slightly improve the model. This is because the lr guides the gradient descent through the error space until the local minimum (or in the optimal case, the absolute minimum) is reached. A high value of the lr causes the network to diverge, while a low value, even though it requires more time, will converge to the local minimum (or in the optimal case, the absolute minimum).

The parameters and results of the training continuation are shown on Table 4.

Observing the retraining results, it is concluded that the model with the best AP is still the one achieved at epoch 7. The lr reduction did not produce the desired effect.

## 4. Text Extraction

### 4.1. Preprocessing

#### 4.1.1. Image Straightening

The VMS image may have a small rotation that affects the OCR. In order to correct it, a procedure based on the Canny algorithm [68,69,70,71] and the Hough transform [69,72,73,74,75] has been designed.

- **Edge detection.** This task is carried out by the Canny algorithm on a grayscale image, on which a 5 × 5 Gaussian filter has been previously applied to reduce noise (although the Canny algorithm already applies one by default). The parameterization used is inspired by [76]. Thresholds are automatically calculated as follows:
  1. Obtain the average pixel intensity, v;
  2. Apply the following formulas with σ=0.33 to find the lower and upper thresholds:
    ○ $T_{l}=\max\left(0,\;\left(1-\sigma \right)\;\cdot \;v\right)$
    ○ $T_{H}=\min\left(255,\;\left(1+\sigma \right)\;\cdot \;v\right)$
- **Straight line recognition within the image.** The Hough transform is applied on the output image of the Canny algorithm, obtaining a list of (ρ, θ) pairs. The parameters established are:
  ○ Accumulator distance on the axis ρ=1;
  ○ Accumulator distance on the axis $\theta =\frac{\pi }{180}\;radians=1{}^{\circ}$;
  ○ Threshold T=100.
- **Calculation of the rotation angle**, θ. For each pair (ρ, θ), Equation (1) is applied to find the equation of the line in the xy plane. From it, the slope, a, required to transform it into degrees using Equation (2) is obtained and entered into a list. The rotation angle, θ, is estimated by the arithmetic mean of all the slopes of the detected lines.
  (1) $$y=\left(-\frac{\cos\theta }{\sin\theta }\right)x+\left(\frac{r}{\sin\theta }\right)$$
  (2) $$degrees=\frac{a\;180}{\pi }$$
- **Calculation of the rotation matrix, *R*.** Finally, by applying a rotation matrix, R (3), to the original image, the straightened image is obtained. For this, it is necessary to calculate α and β by means of Equations (4) and (5), knowing that $center=\left(\frac{width}{2},\frac{altura}{2}\right)$, scale=1 and θ is the value obtained in step three.
  (3) $$R=\left[\begin{array}{ccc} \alpha & \beta & \left(1-\alpha \right)\cdot center\cdot \;x-\beta \cdot center\cdot y\\ -\beta & \alpha & \beta \cdot center\cdot x+\left(1-\alpha \right)\cdot center\cdot \;y \end{array}\right]$$
  (4) α=scale· cosθ
  (5) β=scale·sinθ

#### 4.1.2. Image Cropping

Once the slope has been adjusted, the next step is to crop the image so that only the inside of the VMS is shown. The objective is to identify the lines that delimit the panel and mark the cut points. The following algorithm details the procedure.

1. Find the equations of the lines on the image.

Through steps one, two and three of the above procedure, (ρ, θ) of the horizontal (between 0° and 1° slope), $r_{hi}$, and vertical (between 88° and 92°), $r_{vj}$, lines in the image are obtained. Then, the equations in the xy plane are calculated.

2. Calculate the intersection point with the image limits.

Side limits. For each straight line, $r_{hi}$, the intersection with the vertical limits x=0 (22) and x=w (23), where w is the width of the image, is calculated to store the y coordinate of each slice in the list, $l_{h}$. This way, each element of $l_{h}$ is a candidate to be the limit of the horizontal slice.

(6) $$Left\;side\;cut=\{\begin{array}{c} x=0\\ \;y=a*0+b=b \end{array}$$

(7) $$Right\;side\;cut=\{\;\begin{array}{c} x=w\\ y=aw+b \end{array}$$

Upper and lower limits. For each straight line, $r_{vj}$, the intersection with the horizontal limits y=0 (8) and y=h (9), where h is the height of the image, is calculated to store the x coordinate of each slice in the list, $l_{v}$. This way, each element of $l_{v}$ is a candidate to be the limit of the vertical slice.

(8) $$Upper\;cut=\{\begin{array}{c} \;x=\left(\frac{0-b}{a}\right)\\ y=0 \end{array}$$

(9) $$Lower\;cut=\{\begin{array}{c} \;x=\left(\frac{h-b}{a}\right)\\ y=h \end{array}$$

3. Identify the cutting points and extract the subsection.
  - **Horizontal cut.** Identify the upper, $I_{Hh}$, and lower, $I_{Lh}$, cut-off points of $l_{h}$ that satisfy:
    (10) $$I_{Hh}=\max\left(p\right)\;\mathrm{being}\;p\in l_{h}\;\mathrm{and}\;0\leq p\leq \frac{h}{6}$$
    (11) $$I_{Lh}=\min\left(p\right)\;\mathrm{being}\;p\in l_{h}\;\mathrm{and}\;\left(\frac{5}{6}\;h\right)\leq p\leq h.$$
  - **Vertical cut.** Identify the left, $I_{Lv}$, and right, $I_{Rv}$, cut-off points of $l_{v}$ that satisfy:
    (12) $$I_{Lv}=\max\left(p\right)\;\mathrm{being}\;p\in l_{v}\;\mathrm{and}\;0\leq p\leq \frac{h}{10}.$$
    (13) $$I_{Rv}=\min\left(p\right)\;\mathrm{being}\;p\in l_{v}\;\mathrm{and}\;\left(\frac{9}{10}\;h\right)\leq p\leq h.$$

The range of p values for $I_{Hh}$ and $I_{Lh}$ in addition to $I_{Lv}$ and $I_{Rv}$, as well as the following increments have been experimentally established:

(14) $$I_{Hh}=I_{Hh}+0.03I_{Lh}\;\mathrm{and}\;I_{Lh}=I_{Lh}+0.05I_{Lh}.$$

(15) $$I_{Lv}=I_{Lv}+0.03I_{Rv}\;\mathrm{and}\;I_{Rv}=I_{Rv}+0.05I_{Rv}.$$

#### 4.1.3. Color Adjustment for OCR

Once the VMS content has been isolated, the image is ready for OCR. The objective is to create a new binarized picture, i.e., black text on a white background.

1. **Binarize the image.**
  - **Convert to grayscale.** By applying the formula presented in [77], the gray value is obtained (R, G and B being the values of the red, green and blue channels, respectively).
  - **Apply Otsu’s method.** Otsu binarization [69,78,79,80] is an unsupervised parameterless method that consists of automatically finding a threshold, *T*, that minimizes the intraclass variance in black and white pixels. This way, a binary image is left.
  - **Reverse the image color.** The output of Otsu’s method is an image with white text on a black background. Therefore, it is necessary to apply the NOT logic gate on each value.
2. **Join discontinuous strokes.**

The binarized image may have small discontinuities in the letter strokes. To correct these imperfections that affect recognition, the closing morphological transformation [69,81,82] has been used to solve this problem.

Morphological transformations are operations that usually work on a binarized image by moving a kernel over it (similar to 2D convolution). The closing one (16) consists of a dilation that fills the small holes in the stroke, followed by an erosion that corrects the unwanted pixels that the first operation has enlarged.

(16) A·B=(A⊕B)⊖B

Dilation transforms the value of a pixel to 1 if all pixels below the kernel are 1, and erosion when at least one has the value 1.

3. **Histogram equalization.** Finally, it is necessary to increase the contrast so that the subsequent OCR model will be able to recognize the text. For this purpose, the histogram [69,83] of the image, H(i), has been equalized by mapping it to the normalized cumulative distribution, H’(i), q, which is more uniform.

### 4.2. Recognition and Speech

Once the VMS image has been preprocessed, it is ready to be transcribed using the Tesseract OCR model, and then spoken by the IBM Watson Text to Speech cloud service [25]. Tesseract [84,85,86] is an optical character recognition engine. The version used in this project is Tesseract 4.0, which implements LSTM (long short-term memory) recurrent neural networks, resulting in better and much faster results.

The last step in the pipeline is the voice-over of the content. This task is very easy thanks to the IBM Watson Text to Speech cloud service [25]. It provides the user with a REST API that receives the text and returns an audio file.

## 5. Results and Discussion

The presentation of the results has been divided into two parts, according to the subsystems of the project. All the results have been obtained with the same hardware with which the VMS recognizer model has been trained.

### 5.1. VMS Detector

**Figure 4:**
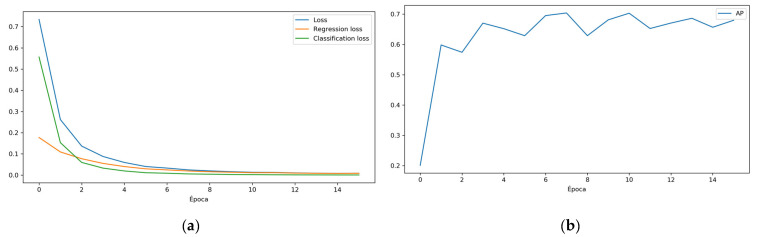
(**a**) Loss, regression loss and classification loss for each training epoch; (**b**) AP by epoch.

An average precision of 0.7 has been achieved on 153 test images. These are some examples of the VMS detector. As can be seen, the detector confuses some static signals as if they were VMSs (Figure 4). This is a reasonable error due to the small number of images used to train the model and the similarity between both types of signals. However, this problem could be solved by adding another machine learning model that classifies between VMSs and non-VMSs. Additionally, different types of VMSs affect processing differently. Basic panels, with road signs and logos on the sides, can be found, as can LED matrices with higher or lower resolutions.

### 5.2. Image Preprocessing and Text Extraction

**Figure 5:**
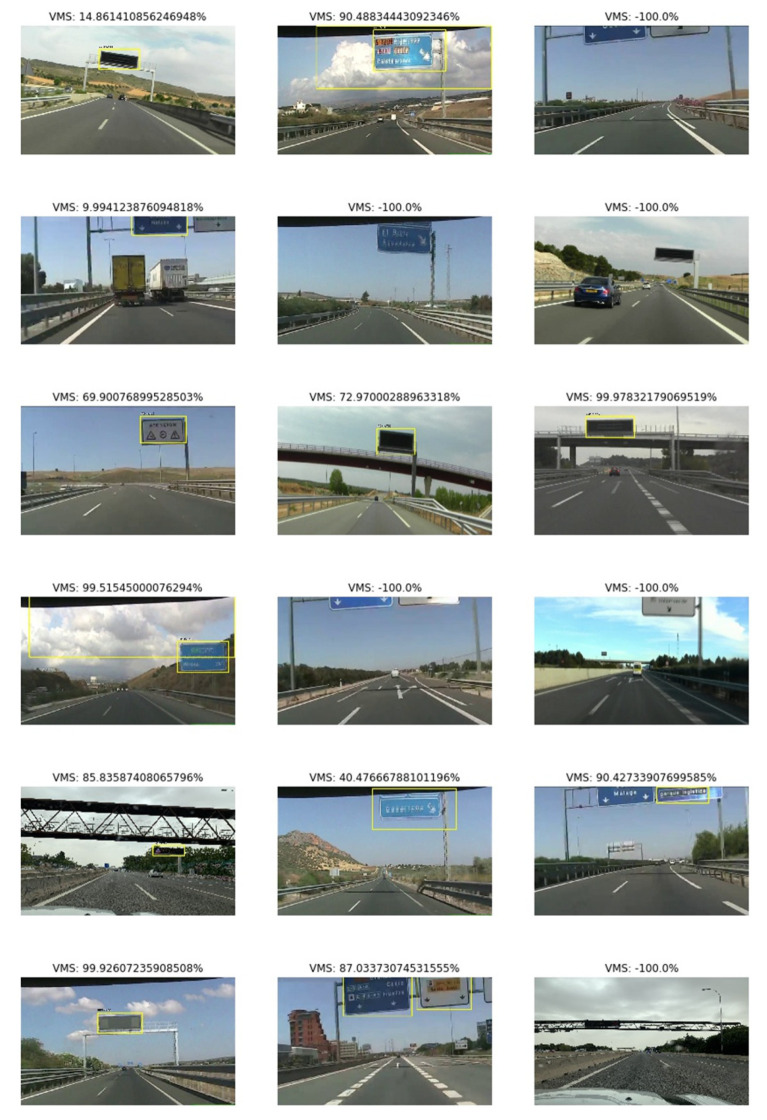
Some examples of VMS detection.

**Figure 6:**
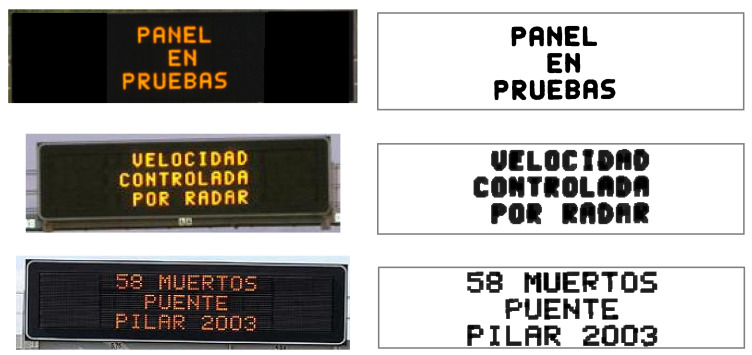

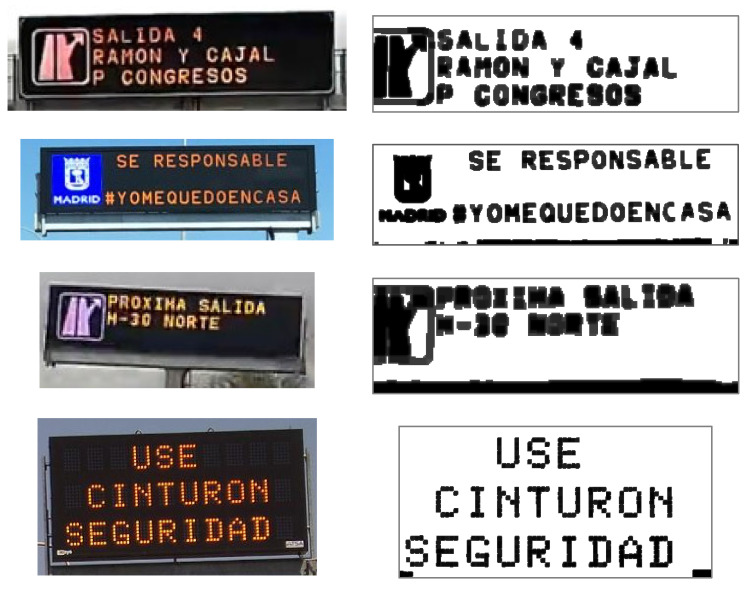
Image preprocessing examples.

Qualitative results of the preprocessing and text extraction are presented below. As can be seen, the quality of the image and the resolution of the VMS affect both preprocessing (Figure 5) and transcription (Figure 6). Images with very low resolution are especially complicated. In addition, signs with pictograms affect the image processing and text extraction in the same negative way.

**Figure 7:**
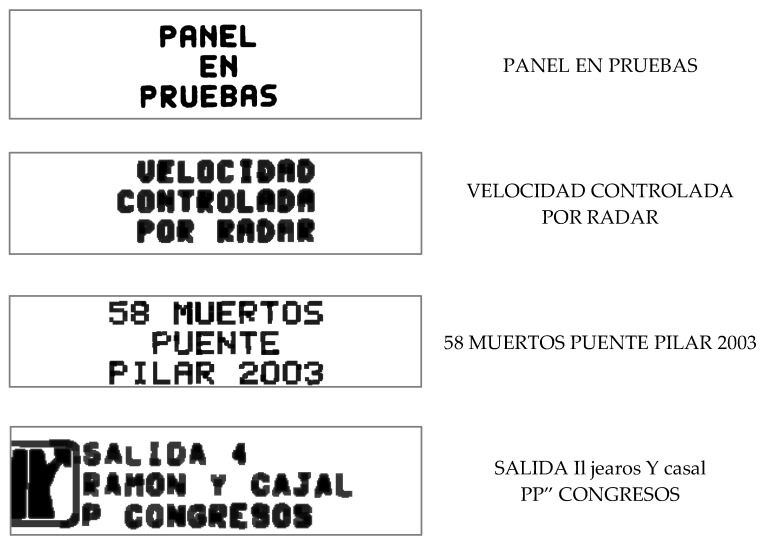

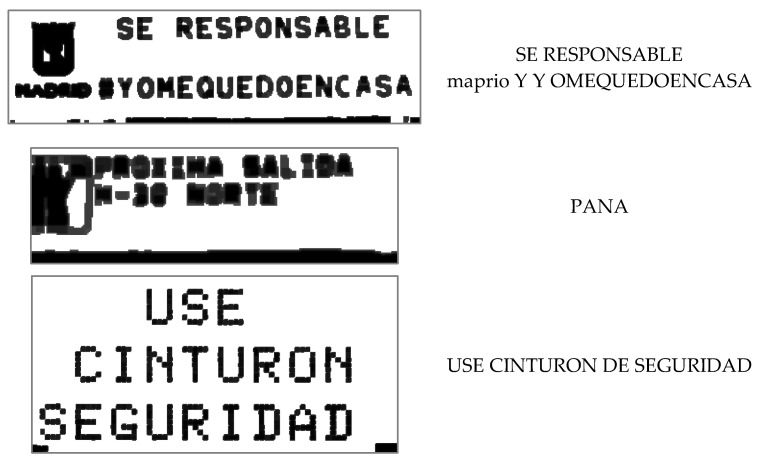
Some examples of OCR (part 1).

**Figure 8:**
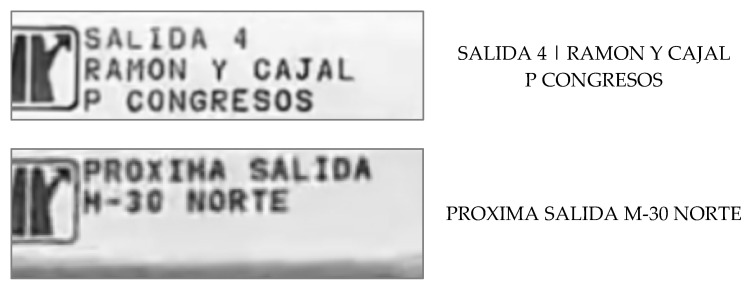
Some examples of OCR (part 2).

It has been detected that, in some images such as the following two (Figure 7), the OCR model performs much better without the last steps of the preprocessing algorithm (Figure 8). In particular, without the last color adjustment, lower resolution instances have a better transcription.

## 6. Conclusions

As a result of the research, a prototype ADAS for reading variable message signs has been obtained. It works with a RetinaNet, a type of neural network based on ResNet50 with an average accuracy of 0.703, which recognizes the VMS in an image and indicates the location of it with a confidence percentage. Next, the section of the image with the VMS is processed to extract the count with an OCR model called Tesseract.

## Figures and Tables

**Table 1:** Basic model training parameters and results.

Epochs	25
No. of training images	134
Time	≈01:30:00
Learning rate	10^−5^
Loss	0.174

**Table 2:** Hardware used for training.

Processor	Intel i7 9800K 3.6 GHz
RAM	32 GBs
Graphics card	Nvidia RTX 2080 Ti
Hard disk	1 Tb SSD M2

**Table 3:** Training parameters and results.

Epochs	16	Best epoch	7
Loss	0.008	Loss (epoch 7)	0.024
lr	10^−5^	AP (epoch 7)	0.703
IoU	0.5	Time	01:20:00

**Table 4:** Retraining parameters and results.

Epochs	14	Best epoch	7
Loss	0.009	Loss (epoch 7)	0.024
lr	10^−7^	AP (epoch 7)	0.703
IoU	0.5	Time	01:15:00

## Appendix A. Dataset Sources

**Table A1:** Sources of the videos used for the preparation of the dataset.

URL	Duration	URL	Duration
youtu.be/MFzuxq4V0XI	00:06:59	youtu.be/dJcH8YFuvY4	00:25:12
youtu.be/GREMRp7rvoY	00:08:16	youtu.be/M2rvG-e04HE	00:16:22
youtu.be/lNhy2mT94Ao	00:05:22	youtu.be/8ifk3BHz1_c	00:13:35
youtu.be/37YgdfidwkA	00:19:58	youtu.be/6bkOZcBECsk	00:12:37
youtu.be/JctnDDdoy0A	00:22:56	youtu.be/afCyj52txC0	00:06:40
youtu.be/H1gxWeWsa_E	01:20:25	youtu.be/vU82-jnUi_E	00:07:57
youtu.be/UZFLDp_LLj4	00:27:23	youtu.be/D5RHKJNhw7I	00:08:01
youtu.be/tz8bEIirIx4	00:10:50	youtu.be/S1DE3pvnG8s	00:12:33
youtu.be/QJ_XSlOeCBw	00:10:48	youtu.be/XLQbclKjNrw	00:03:36
youtu.be/4s-WfvYUbPM	00:19:57		
